# Subserosal hematoma of the sigmoid colon after vaginal delivery

**Published:** 2017

**Authors:** N Bacalbașa, RE Bohîlțea, M Dumitru, N Turcan, MM Cîrstoiu

**Affiliations:** * “Carol Davila” University of Medicine and Pharmacy, Bucharest, Romania; University Emergency Hospital Bucharest, Romania

**Keywords:** hematoma, sigmoid colon, vaginal delivery complication

## Abstract

Postpartum hemorrhage is an obstetrical emergency that represents the leading cause of maternal mortality. Severe hemorrhagic complications that could appear postpartum are the abdomino-pelvic hematomas, which result from the rupture of the pelvic vessels. We reported a very rare case of puerperal retroperitoneal subserosal hematoma of sigmoid colon following vaginal delivery, which was successfully managed by conservative methods. As far as we know, there are only a few case reports of intramural hematoma of sigmoid colon in literature, having other etiologies than vaginal delivery trauma. The particularities of the case consisted in the association of hemangiomas and the low risk thrombophilia. Diagnosis was based on the clinical exam and the paraclinical founding. Laparotomy is generally considered the last choice, in life threatening cases with hemodynamic instability, compression signs, and presence of contrast leakage on noninvasive imaging methods, but avoiding colonic resection after dissection represented the true challenge of the case.

## Introduction

It is known that labor and implicitly delivery include a broad spectrum of possible complications, postpartum hemorrhage being one of the most common and also one of the major causes of maternal death [**1**]. The challenge that comes with the postpartum hemorrhage is appreciating the volume of bleeding from where it becomes abnormal and recognizing the first signs of hemorrhagic shock as quickly as possible. The dominant cause of primary postpartum hemorrhage is uterine atony, being followed by cervical, vaginal, and perineal lacerations, retained placental tissues, abnormal placentation and coagulopathy [**2**]. Rare but severe hemorrhagic complications that could appear postpartum are the abdomino-pelvic hematomas, which result from rupture of the pelvic vessels [**3**]. Classically, puerperal hematomas are divided according to location, into vulvar, vulvovaginal, vaginal and subperitoneal [**4**]. All puerperal hematomas represent obstetric life-threatening emergencies, usually the extension and the volume being directly related with the parturition aspects. They become clinically apparent within 24 hours of delivery and in the majority of cases involve the branches of hypogastric artery, vestibular bulbospongiosus body’s arteries, or branches of pudendal artery: rectal inferior arteries, perineal and clitoridean arteries [**5**]. Their association with the perineal lacerations, episiotomy or instrumental assisted deliveries, is common. Arteriovenous malformations have been reported in association with hematoma formation. In rare situations, when the vascular damage is extended above the pelvic fascia, the result could be the forming of hematomas located over the levator ani muscles that can dissect and extend retroperitoneally trough retrocolic area up to the hepatic angle and the lower edge of the diaphragm, in the process of extension.

The clinical presentation includes a variety of unspecific symptoms: from abdominal pain to signs of a significant loss of blood, tachycardia, palpitations, paleness and a generally unwell status.

As far as we know, we reported a very rare case of puerperal retroperitoneal subserosal hematoma of sigmoid colon following vaginal delivery, which was successfully managed by conservative methods.

## Case report

**Fig. 1:**
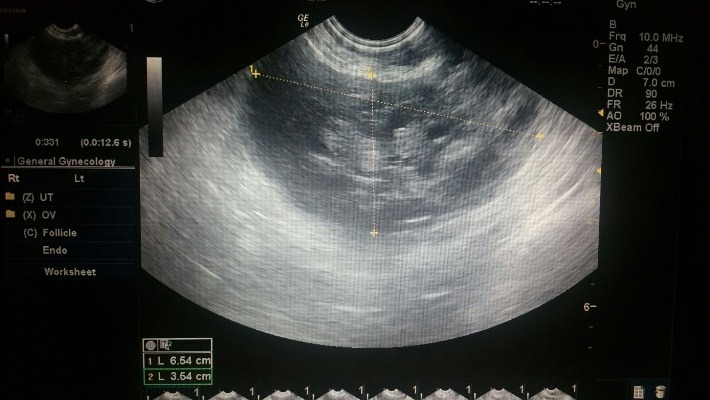
Ultrasound image of postpartum perirectal hematoma

A 27-year-old woman (gravida 1, para 1) was admitted to the labor unit of our hospital due to the spontaneous rupture of the membranes and painful uterine and regular contractions at 40 weeks’ gestation. The course of the present pregnancy was uneventful, with insignificant family medical history and without personal pathological antecedents except for hemangiomas, located at the level of the liver and superior lip, chronic constipation, and homozygous mutation of PAI genes, detected after a first trimester screening, that revealed a high resistance in the uterine artery flow, followed by low-dose Aspirin administration until 36 weeks of gestation. Clinical exam revealed a 3 cm dilatation locally, cranial applied presentation with loss of opalescent amniotic fluid. Paraclinical, on admission, the value of hemoglobin was 13.2 g/ dl, white blood cells count 14.02*103/ µl and normal parameters of coagulation with no signs of genital or urinary tract infection. Labor progressed relatively precipitated, the patient spontaneously delivering a 3300 grams boy with the 1-minute Apgar score 10, within 3 hours and 30 minutes. A third (3a) degree perineal rupture resulted in moderate vaginal bleeding. The placenta was completely delivered with minimal blood loss. After the suture of the perineal rupture and the provided hemostasis, the patient presented a good general condition and a normally contracted uterus with no abnormal blood loss. Two hours after the delivery, the patient complained of severe pelvic and low abdominal pain, palpitations, sweating, and tachycardia. On examination, the teguments appeared pale, the uterus well contracted with no excessive vaginal bleeding. On palpation, the abdomen was soft, without any signs of peritoneal irritation, but vaginally posterior and proximal; a tender formation was palpable ascending the cervix. The presence of the palpable hypoechogenic perirectal 6,5/ 3,5cm structure was objectified on ultrasound, suggesting the diagnosis of perirectal hematoma (**Fig. 1**). 

**Fig. 2:**
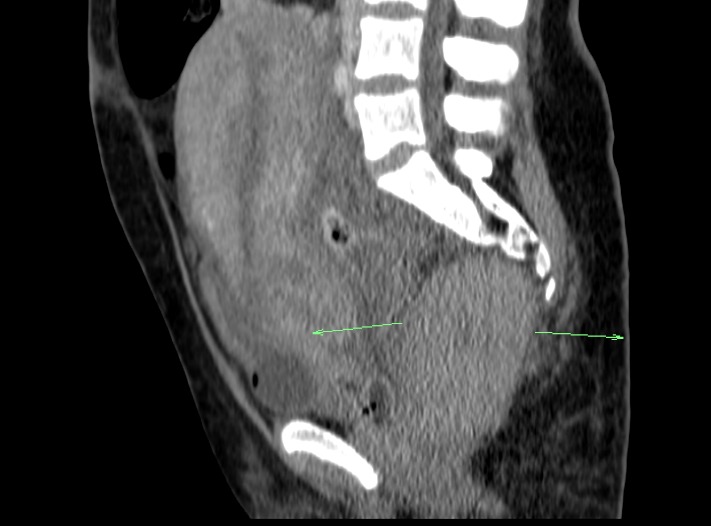
CT image of retro rectal and pelvic retroperitoneal hematoma fused from a perineal laceration associated with childbirth

Paraclinical, the value of hemoglobin was 9.9 g/ dl and white blood cells 35.8 *103/ µl. The urine was clear on the urinary probe. A surgical reintervention was decided with the dissolution of the past suture, the eviction of the hematoma, drainage and the reapproximation of the laceration area. Post operatory, the patient was admitted in the intensive therapy unit, with the constant monitoring of the vital parameters, successive blood analyses and a large spectrum antibiotic therapy being instituted. Despite the expectations, the general status of the patient continued to deteriorate. A computerized tomographic (CT) scan revealed the presence of a retro rectal and pelvic retroperitoneal hematoma with the axial dimensions of 102/ 65 mm and craniocaudal diameter of 100 mm, with a non-iodophilic, clear delimitation from surrounding organs, and no evidence of active perineal or pelvic bleedings. Intraperitoneally, there seemed to be a significant quantity of fluid, predominantly pelvic and minimally perihepatic disposed, with no sanguine density aspects (**Fig. 2**).

**Fig. 3:**
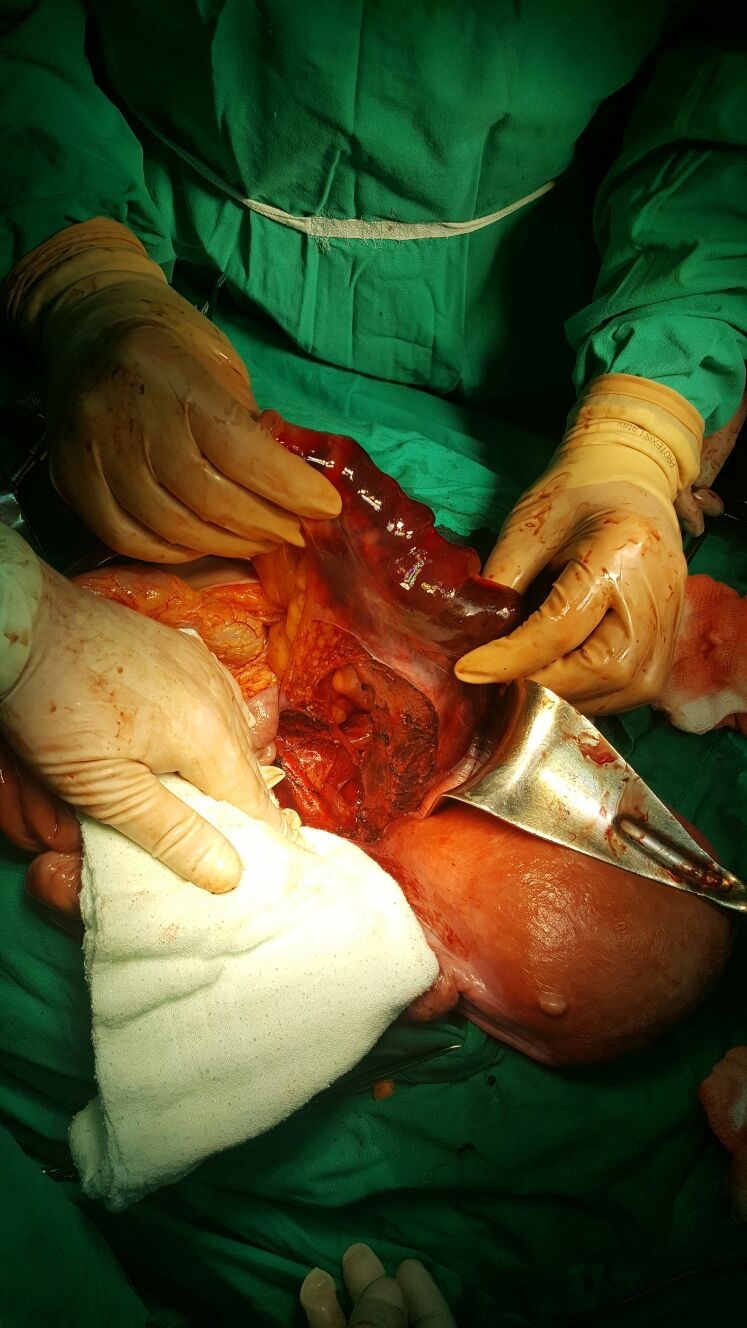
Macroscopic necrotic aspect of sigmoid colon

**Fig. 4:**
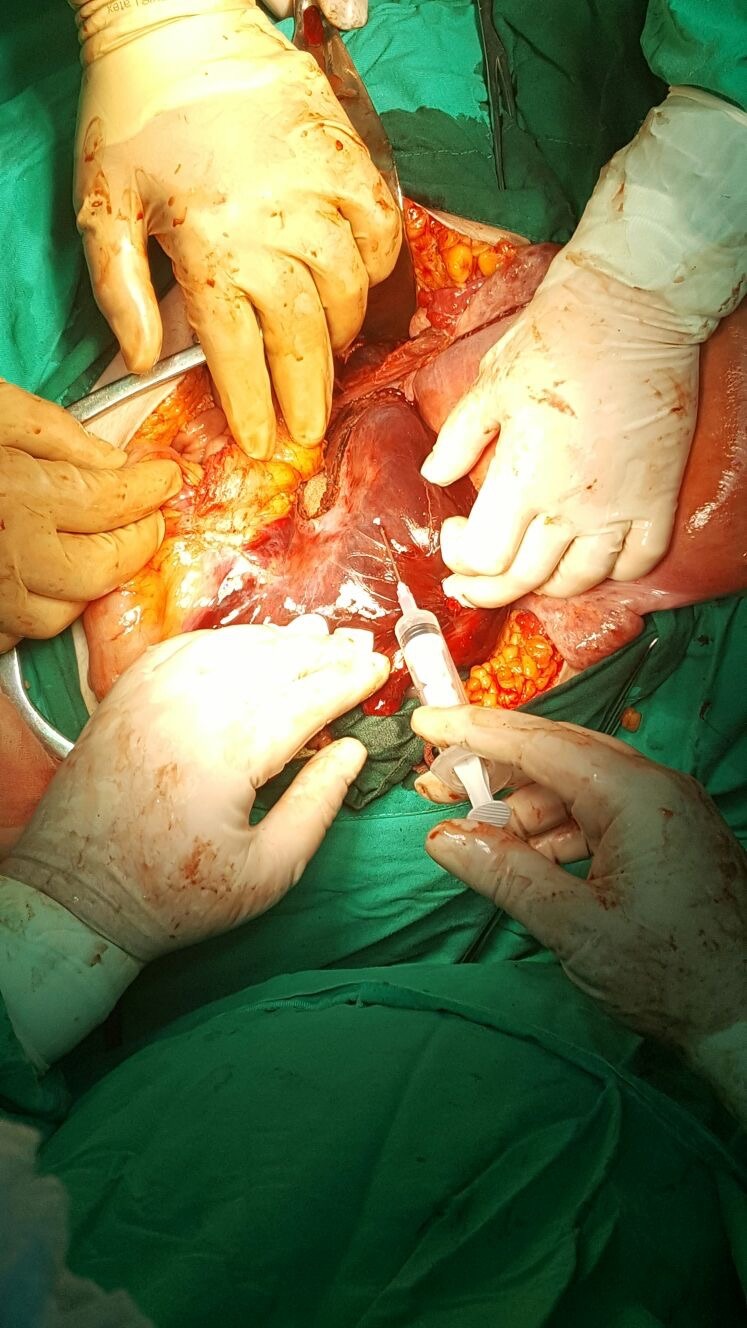
Repeated intramural lidocaine hydrochloride 1% injections of sigmoid

Ten hours post intervention, the laboratory’s findings included a decreasing value of hemoglobin, of 6.4 g/ dl, white blood cells count of 21.0*103/ µl, platelets count of 79*103/ µl, creatine kinase isoenzymes (CKI) of 687 U/ L superimposed on the patient’s general state of illness; blood pressure was of 140/ 80 and her pulse was of 110 bpm. An emergency diagnostic and therapeutic laparotomy was decided and applied. Intraoperatively, a retroperitoneal retro rectal hematoma was noted, extended through the mesosigmoid under the sigmoid colon serosa. After the dissection of sigmoid and rectum nearby pelvis floor without finding any active source of bleeding, despite the general necrotic aspect of these segments (**Fig. 3**), an expectant attitude was decided considering the positive response of the intestinal wall on the instillation of lidocaine hydrochloride 1% and repeated cold peritoneal lavage (**Fig. 4**).

The surgery had an uneventful course, the patient being hemodynamically and respiratory stable all the time. The patient received sanguine transfusions, platelet mass, large spectrum antibiotic therapy, pain relievers, and also anti-inflammatory therapy in the intensive care unit. The evolution was favorable during the next days, transit for gases being resumed in the following 24 hours and for faeces in the next 48 hours. The patient was discharged in 10 days after the intervention, the sonographic evaluation of the pelvis prior to discharge revealing a normal-looking uterus and ovaries with no findings suggesting the presence of a residual hematoma. 

## Discussions and conclusions 

Postpartum hemorrhage is an obstetrical emergency representing the leading cause of maternal mortality [**6**]. Vaginal or cervical lacerations are responsible for 2% of the cases of postpartum hemorrhage [**7**]. Zahn et al. and Sheikh affirmed that nulliparity is a strong risk factor for vaginal hematoma [**8**]. Non-invasive methods are preferred when a puerperal hematoma is suspected. The first investigation line is ultrasonography, with an accurate diagnosis of pelvic and abdominal hematomas. More complex cases require a CT scan with an arterial phase, that can elucidate the suspicion of an active bleeding [**9**].

Regarding the management, endovascular treatment is preferential when the source of the bleeding is arterial and obvious and the state of the patient is hemodynamically stable. The indications for laparotomy are hemodynamic instability, compression signs, and presence of contrast leakage on noninvasive imaging methods [**10**]. 

Retroperitoneal and retrorectal hematomas are uncommon following vaginal delivery, a more uncommon complication of labor being the extinction of the hematoma through the retroperitoneal pathway into the intestinal wall. Diagnosis is based on the clinical exam and paraclinical founding. Laparotomy is considered the last choice, in life threatening cases. 
